# Mixed method versus full top-down microcosting for organ recovery cost assessment in a French hospital group

**DOI:** 10.1186/s13561-016-0133-3

**Published:** 2016-11-28

**Authors:** Abdelbaste Hrifach, Coralie Brault, Sandrine Couray-Targe, Lionel Badet, Pascale Guerre, Christell Ganne, Hassan Serrier, Vanessa Labeye, Pierre Farge, Cyrille Colin

**Affiliations:** 1 Hospices Civils de Lyon, Pôle Information Médicale Evaluation Recherche, Unité d’Evaluation Médico-Economique, 162, avenue Lacassagne - Bâtiment A, 69424 Lyon, Cedex 03 France; 2 Département d’Information Médicale, Hospices Civils de Lyon, Pôle Information Médicale Evaluation Recherche, 69424 Lyon, France; 3 Hospices Civils de Lyon, Hôpital Edouard Herriot, Service d’Urologie, 69437 Lyon, France; 4 Hospices Civils de Lyon, Cellule Innovation, Délégation à la Recherche Clinique et à l’Innovation, 69237 Lyon, France; 5 Hospices Civils de Lyon, Hôpital Edouard Herriot, Coordination Hospitalière de Prélèvement d’Organes et de Tissus, 69437 Lyon, France; 6 Université Claude Bernard Lyon 1, 69008 Lyon, France; 7 Univ. Lyon, Université Claude Bernard Lyon 1, HESPER EA 7425, F-69008 Lyon, France

**Keywords:** Top-down, Bottom-up, Microcosting, Organ recovery

## Abstract

**Background:**

The costing method used can change the results of economic evaluations. Choosing the appropriate method to assess the cost of organ recovery is an issue of considerable interest to health economists, hospitals, financial managers and policy makers in most developed countries.

**Objectives:**

The main objective of this study was to compare a mixed method, combining top-down microcosting and bottom-up microcosting versus full top-down microcosting to assess the cost of organ recovery in a French hospital group. The secondary objective was to describe the cost of kidney, liver and pancreas recovery from French databases using the mixed method.

**Methods:**

The resources consumed for each donor were identified and valued using the proposed mixed method and compared to the full top-down microcosting approach. Data on kidney, liver and pancreas recovery were collected from a medico-administrative French database for the years 2010 and 2011. Related cost data were recovered from the hospital cost accounting system database for 2010 and 2011. Statistical significance was evaluated at *P* < 0.05.

**Results:**

All the median costs for organ recovery differ significantly between the two costing methods (non-parametric test method; *P* < 0.01). Using the mixed method, the median cost for recovering kidneys was found to be €5155, liver recovery was €2528 and pancreas recovery was €1911. Using the full top-down microcosting method, median costs were found to be 21–36% lower than with the mixed method.

**Conclusion:**

The mixed method proposed appears to be a trade-off between feasibility and accuracy for the identification and valuation of cost components when calculating the cost of organ recovery in comparison to the full top-down microcosting approach.

**Electronic supplementary material:**

The online version of this article (doi:10.1186/s13561-016-0133-3) contains supplementary material, which is available to authorized users.

## Background

Choosing the appropriate costing method to estimate accurate costs in healthcare is an issue of considerable interest to health economists for economic evaluations, to hospitals for financial management purposes, and to policy makers in most European countries.

Conventional costing methods are mainly characterized by how the two main cost components, i.e., the process of identification and the process of valuation, are ordered. Four standard costing methods have been developed that use microcosting or gross costing for identification and top-down or bottom-up for valuation [1]. For cost identification, in microcosting, all cost components are defined at the most detailed level, whereas in gross costing, cost components are defined at a highly aggregated level. For cost valuation, in the bottom-up approach, cost components are valued by identifying resources used directly employed for a patient, whereas in the top-down approach, cost components are valued by separating out the relevant costs from comprehensive sources. Combining these dimensions gives four theoretic approaches: top-down gross costing, top-down microcosting, bottom-up gross costing and bottom-up microcosting. Some cost differences are simply due to differences in costing methodology and reflect different levels of accuracy. Previous publications have concluded that the costing method selected significantly changes the results of economic evaluations [2, 3]. Whereas bottom-up microcosting is considered to overestimate assessed costs, top-down gross costing tends to underestimate them [1].

Regarding healthcare, this methodological aspect is even more important when cost evaluations cover different countries. In the past, major variations in hospitals’ cost accounting systems have been identified across Europe, making comparisons meaningless [4]. Negrini et al. highlighted the need for a standardized method for all European countries to accurately measure the costs of hospitals’ activities [5]. The introduction of Diagnosis Related Groups in Europe and the development of activity-based pricing offered the opportunity, in most European countries, to develop medico-administrative and economic databases to analyse hospital activities with a standardized cost-accounting system [6]. This convergence in the structural organization of European databases allows comparisons in terms of resource consumption between countries and the development of costing methods that can address healthcare issues common throughout Europe [7].

The internationalization of health issues is particularly advanced in the field of organ recovery and organ transplantation [8–10]. Seven European countries rank in the top 10 countries worldwide in practicing organ recovery from deceased donors according to the International Registry in Organ Donation and Transplantation [11]. The improved survival rates obtained with transplantation and the shortage of available organs have made organ procurement a vital challenge across Europe [12, 13]. Given the limited resources for healthcare provision, organ recovery cost data remains paradoxically scarce in most European countries. In the future, the increasing number of patients requiring organ transplants in Europe will make organ recovery cost an international public health issue [14]. For these reasons, organ recovery appears to be an interesting topic on which to elaborate and test an alternative to conventional costing methods.

Bottom-up microcosting is considered to be the best method to estimate hospital services’ costs, but it is known to consume resources and time [15]. Drummond et al. recommended using the bottom-up microcosting method to evaluate cost components that have a great impact on total costs [16]. Discussions with the hospital coordination of organ and tissue procurement allowed us to elaborate the working hypothesis that consumables are the most expensive item out of all organ recovery costs. Instead of conducting a full bottom-up microcosting approach during a multi-step emergency procedure that compromises real-time acquisition of cost data, we developed a mixed method combining top-down microcosting and bottom-up microcosting to accurately assess the costs of organ recovery.

### Objectives

The main objective of this study was to compare a mixed method, combining top-down microcosting and bottom-up microcosting, versus full top-down microcosting to assess the cost of organ recovery in a French hospital group. The secondary objective was to describe the cost of kidney, liver and pancreas recovery from French databases using the mixed method.

## Methods

### Study design

The study was implemented in the hospital group the ‘*Hospices Civils de Lyon’* (HCL) from January 2010 to December 2011. The HCL acts as a tertiary referral centre for organ recovery and organ transplantation. Direct medical costs were estimated from the hospital’s perspective because organ recovery costs come from hospital budgets. Direct non-medical costs and indirect costs were not considered in this study. The timeframe considered was from the diagnosis of brain death until completion of the organ recovery procedure. The costs related to family management, machine perfusion and organ shipment were not included because they are not directly related to the organ recovery procedure.

### Registers used

In France, a national hospital discharge database and a hospital cost accounting system were developed. Their use, as a medico-administrative and an economic database, respectively, allowed for carrying out previous medico-economic analyses [17]. The French College of Health Economists recommended using this medico-administrative database for health economic evaluations [18].

The hospital cost accounting systems implement a national economic database that groups together representative hospitals using top-down microcosting [19]. The HCL group collected cost data during the years 2010 and 2011 through the hospital cost accounting systems. For each discharge abstract collected for each hospital-stay data on medical procedures, all the resources consumed were identified, quantified and valued using the following methodologies.

### Patients and organ collection

All actual brain death donors who underwent kidney, liver or pancreas recovery in the HCL between 2010 and 2011 were eligible for the analysis. Kidney recovery in this paper systematically refers to the removal of both kidneys. Donors were excluded if cost and/or hospital-stay data were unavailable. Actual donors were identified in a medical database and selected using the Common Classification of Medical Procedures codes corresponding to the recovery of kidneys, liver and pancreas from donors after brain death. Living donors and donors after circulatory death were excluded.

### Identification of cost components

The resource identification started from the para-clinical examinations to determine brain death to the completion of the organ recovery procedure. The microcosting approach made it possible to identify, from discharge abstracts and discussion with hospital coordination of organ and tissue procurement, seven items divided into 39 sub-items. The items were surgery, anaesthesia, intensive care, logistics, biology, imaging and consumables. The sub-items have been detailed in Additional file 1: Table S1 of the Supplementary Digital Content (SDC).

### Valuation of cost components

All cost components were valued in 2011 Euros. In the mixed method, surgery, anaesthesia, intensive care, logistic, imaging and biology were valued using a top-down approach while consumables were valued using a bottom-up approach. In the full top-down microcosting method, all items were valued using a top-down approach (Table 1).

**Table 1:** Identification and valuation modalities of the seven items analysed in the mixed method

Items	Identification	Valuation
Surgery	Microcosting^a^	Top-down^c^
Anaesthesia	Microcosting^a^	Top-down^c^
Intensive Care	Microcosting^a^	Top-down^c,d^
Logistics	Microcosting^a^	Top-down
Imaging	Microcosting^b^	Top-down^e^
Biology	Microcosting^b^	Top-down^f^
Consumables	Microcosting^b^	Bottom-up^g^

^a^Discharge abstracts

^b^Hospital Coordination

^c^Relative Cost Index

^d^Length of Stay

^e^Common Classification of Medical Procedure

^f^Nomenclature of Medical Biology Procedures

^g^Negotiated Price for HCL in 2011

Surgery, anaesthesia and intensive care items were valued from the HCL cost accounting weighted, respectively, by the surgery Relative Cost Index (RCI), anaesthesia RCI and a combination of the anaesthesia RCI and length of stay. The RCI is used to assess the cost of a procedure carried out in ideal conditions [20]. It is used to break down the overall cost according to the RCI number specific to each procedure [21].

Logistics items (sterilization, biomedical engineering, hygiene and vigilance) were valued from the HCL cost accounting by isolating components specifically related to organ recovery.

Biology and imaging exams were valued in the context of multiple organ recovery. This valuation was based on the Common Classification of Medical Procedure, Nomenclature of Medical Biology Procedures and the Directory of establishments receiving global allocation funding [22]. Biology testing and imaging costs were weighted by the number of organs recovered from each donor.

For the mixed method analysis, consumables were valued using a bottom-up approach through the unit purchase price for the HCL. As the consumables required for kidney and pancreas recovery are provided in the same surgical kit, their costs were wholly attributed to kidney procurement or divided between kidney and pancreas procurement according to which organs were actually recovered (SDC, Additional file 1: Table S2, Cost of surgical kit for kidney recovery in the HCL and Additional file 1: Table S3, Cost of surgical kit for pancreas recovery in the HCL). As the consumables needed for liver recovery are provided in a separate surgical kit, the costs were valued using the same methodology and wholly attributed to liver recovery (SDC, Additional file 1: Table S4, Cost of surgical kit for liver recovery in the HCL). The preservation fluid required for each organ was also valued from the HCL purchase price.

For the full top-down microcosting analysis, consumables were also valued using a top-down method according to the HCL cost accounting system.

### Outcomes

#### Comparison of the mixed method versus full top-down microcosting

The results of the mixed method were compared using the Wilcoxon signed-rank test for two samples to those obtained from the full top-down microcosting approach to investigate possible differences (Table 2). Organ recovery costs were presented according to their median and interquartile range (IQR). Statistical significance was evaluated at *P* < 0.05.

**Table 2:** Mixed method versus full top-down microcosting to assess organ recovery cost in HCL (2011 Euros)

			Mixed Method		Full Top-down Microcosting			
Organ(s)	Organ(s)	*N*	Median	IQR	Median	IQR	P*	∆Median (%)
*1* ^*st*^ *evaluation*								
	Kidneys	83	5,155	913	3,283	1,428	˂0,0001	−36,32
	Liver	63	2,528	447	1,826	1,138	˂0,0001	−27,78
	Pancreas	25	1,911	208	1,505	322	0,0127	−21,26
*2* ^*nd*^ *evaluation*								
	Kidneys alone	21	5,439	710	3,811	2,139	0,0001	−29,93
	Kidneys + Liver	27	8,060	944	5,830	2,665	˂0,0001	−27,66
	Kidneys + Liver + Pancreas	23	8,562	766	5,822	1,225	˂0,0001	−32,00
*3* ^*rd*^ *evaluation*								
	Kidneys alone	21	5,439	710	3,811	2,139	0,0001	−29,93
	Kidneys + 1 other organ	29	5,436	677	3,639	1,229	˂0,0001	−33,06
	Kidneys + 2 or more other organs	33	4,551	631	2,910	528	˂0,0001	−36,05

p*: Wilcoxon signed-rank test

Three cost evaluations of organ recovery were conducted using SPSS Statistics 19. Each explores a specific characteristic of organ recovery procedures.

1. The cost of separate kidney, liver and pancreas recovery regardless of the organs actually recovered to obtain the cost for each organ recovered with a view to future medico-economic studies.
  2) The cost of the most common organ recovery procedures: kidneys alone, kidneys and liver, kidneys, liver and pancreas to better reflect the realities of organ recovery.
    3) The cost of kidney recovery according to the number of organs recovered to highlight a possible economy of scale when several organs are recovered simultaneously.

#### Analysis of organ recovery costs calculated by mixed method

The relative proportion of expenditure items was also specified for separate kidney, liver and pancreas recovery to test the initial hypothesis regarding the high proportion of consumable costs with regard to the total cost (Table 3). Moreover, we analysed the cost of kidney recovery according to the number of recovered organs to highlight potential economies of scale.

**Table 3:** Breakdown of mean cost assessed by mixed method for the recovery of kidneys, liver and pancreas in the HCL (2011 Euros)

Cost Euros (%)								
ORGAN	Surgery	Anaesthesia	Intensive Care	Logistics	Imaging	Biology	Consumables	Total
Kidneys	750 (15%)	676 (13%)	697 (13%)	95 (2%)	202 (4%)	359 (7%)	2,356 (46%)	5,135 (100%)
Liver	227 (9%)	284 (11%)	318 (13%)	51 (2%)	162 (6%)	287 (11%)	1,244 (48%)	2,574 (100%)
Pancreas	224 (12%)	283 (15%)	194 (10%)	40 (2%)	105 (5%)	187 (10%)	894 (46%)	1,928 (100%)

## Results

93 actual brain death donors underwent kidney, liver or pancreas recovery in the HCL. 2 donors, corresponding to 2 pairs of kidneys, were excluded due to missing cost data. A total of 91 actual brain death donors were included in the analysis corresponding to a total of 83 pairs of kidneys, 63 livers and 25 pancreases. The organs recovered but not analysed were lungs, heart and intestines (SDC, Additional file 1: Table S5, Organs recovered in the HCL from January 2010 to December 2011).

### Comparison of the mixed method versus full top-down microcosting

The cost of organ recovery assessed using the mixed method differs significantly from the cost obtained using the full top-down microcosting approach (Wilcoxon signed-rank test for two samples; *P* < 0.01).

Using the mixed method, the median cost for kidneys, livers and pancreases was assessed as €5155 (IQR €913), €2528 (IQR €447) and €1911 (IQR €208), respectively. The cost of the following combinations: kidneys alone; kidneys and liver; and kidneys, liver and pancreas was assessed at €5439 (IQR € 710), €8060 (IQR €944) and €8562 (IQR €766), respectively.

Using the full top-down microcosting approach, the median cost fell by 21–36% compared to the mixed costing method, and the interquartile range increased considerably. Thus, the median costs for kidneys, livers and pancreases were assessed as €3283 (IQR €1428), €1826 (IQR €1138) and €1505 (IQR €322), respectively. The cost of the following combinations: kidneys alone; kidneys and liver; and kidneys, liver and pancreas was assessed at €3811 (IQR €2139), €5830 (IQR €2665) and €5822 (IQR €1225), respectively. In comparison with the mixed method, the median costs are lower using the full top-down method and the interquartile ranges are wider.

### Analysis of organ recovery costs calculated by mixed method

The mean costs assessed by the mixed method for recovery of kidneys, liver and pancreas in the HCL showed that the consumables were the largest item in terms of value and proportion regardless of the organ analysed (Table 3). They accounted for almost half of the total cost. Out of the different consumables, the preservation fluid was the most expensive item (€364 for 2 l) (SDC, Additional file 1: Table S2, Cost of surgical kit for kidney recovery in the HCL). Surgery, anaesthesia and intensive care items represented approximately one-third of the total cost in each case. The remaining cost, between 13 and 19%, included logistics, imaging and biological testing (Table 3).

Moreover, the cost of kidney recovery according to the number of recovered organs was assessed at €4551 (IQR €631) (*N* = 33) and €5439 (IQR € 710) (*N* = 21) in the HCL (Table 2). This cost gradually decreased as the number of organs recovered increased. Surgery, anaesthesia, intensive care and logistics have approximately constant values. The cost reduction was related to the gradual decrease in the cost of consumables, imaging and biological testing due to economies of scale.

## Discussion

The mixed method makes it possible to assess organ recovery cost from French databases and appears to combine the accuracy of the bottom-up method and the simplicity of the top-down approach. The costs of organ recovery assessed using the mixed method differ significantly from those assessed using the full top-down microcosting approach. The full top-down microcosting approach resulted in a 21–36% lower median cost and carried the risk of underestimating assessed costs in comparison to the mixed method. There are several explanations for the differences between the two methods. The full top-down method gives all items identified the same degree of accuracy regardless of the importance of the different cost components in terms of value and proportion. Although consumables represent the largest part of the cost of organ recovery, the full top-down method cannot include all consumables because they are not adequately tracked at the patient level. The fact that consumables represent 46% of the total cost confirms the working hypothesis to use a bottom-up strategy to value this item.

The validation of the proposed methodology through the comparison of our results to national and international cost data cannot be carried out because the available cost data are scarce and old. Currently, few European countries have examined organ recovery costs from a hospital perspective. Lenisa et al. assessed the cost of kidney and pancreas procurement at $1400 in the Italian healthcare system [23]. A single publication assessed the cost of pancreas procurement in the French system at €1086 [24]. In Spain, a cost-benefit estimation found kidney procurement to be $3162 [25]. Although tariffs are different from costs, being unable to compare our results to recent costs, we compared the results of this analysis to the French tariffs. The specific tariffs (PO1 to PO4) established for hospital remuneration for organ recovery activities in the French healthcare system are closer to the estimates made with the mixed method than the estimates made with the full top-down method [26]. Under the 2011-PO3 tariff (€8473), the fee for simultaneous kidney, liver and pancreas recovery is closer to the estimate made through the mixed method (€8562) than the estimate made with the full top-down method (€6180). This information seems to indicate a better estimation of organ recovery valuation with the mixed method in comparison to the full top-down approach.

Some limitations of our work should be noted. From a methodological point of view, the valuation of the surgery and anaesthesia items needs to be improved. Both items were assessed using relative cost index, which does not reflect the actual time spent by surgeons and anaesthetists on the procedure. Therefore, neither the full top-down method nor the proposed mixed method accurately measures these variables. As suggested by Mercier et al., the introduction of time-driven activity-based costing will provide a more accurate weighting, with minimal adjustment, while offsetting the non-consideration of operation time variation in top-down methods [27]. Moreover, considering indirect costs, such as overhead and capital, would contribute to a more comprehensive cost estimation for hospital services [28]. Although the hospitals participating in the national economic database use a common top-down microcosting procedure for calculating costs, some variations in the practical implementation were observed that cannot be analysed from a single hospital group. Extending the proposed mixed methodology to a larger number of European hospitals would offer methodological and financial results. Cost data from a limited number of hospitals in several countries would be preferable to analysing a large number of hospitals in one country [29].

Extrapolation of the mixed method to other countries requires the availability of both a cost accounting system and a medico-administrative database. Moreover, only countries using a top-down microcosting approach for direct cost allocation such as Germany, England and Estonia will allow the implementation of the mixed method proposed in the French context [4]. The mixed method appears to be difficult to extrapolate to countries using a full bottom-up costing approach such as Finland, the Netherlands and Sweden [4]. However, it would be interesting to compare the results of the mixed method with those of countries using a full bottom-up method. Effectively, comparisons of the value and the proportion of the seven items identified for organ recovery would indicate if the mixed method provides sufficient accuracy when compared to the full bottom-up method. According to Geissler et al., Ireland, Portugal and Spain have low-quality patient-level cost information [6]. Extrapolating the mixed method to these countries would also be interesting. The use of a mixed methodology seems to be effective when conducting cost studies in countries with limited or low-quality data [30].

## Conclusions

Through the procedure of organ recovery, this study highlights the necessary trade-off between top-down simplicity and bottom-up accuracy to obtain valid cost estimates to meet the increased demand for medico-economic data. It would be interesting to test the mixed method on other medical procedures. The proposed method could be used whenever a potentially expensive technological innovation is introduced into a previously well valued procedure in order to focus the valuation efforts on the unknown portion of the cost. In the same way, the mixed method could also be used for a not yet valued procedure when a cost component is known to represent a large part of a total cost. In all these situations, the mixed method can provide feasibility, rapidity and accuracy.

### Supplementary data

Supplementary data are available online.

## Key-points

- The mixed method appears to be a trade-off between the accuracy of the bottom-up approach and the simplicity of the top-down approach.
- The cost of organ recovery assessed using the mixed method differs significantly from the cost obtained using the full top-down microcosting approach.
- The use of a full top-down microcosting method carries the risk of underestimating assessed costs in comparison to the mixed method.
- Extrapolation of the mixed method to other countries requires the availability of both a cost accounting system and a medico-administrative database.
- The mixed method could be used whenever an expensive technological innovation is introduced into a previously well valued procedure or for a not yet valued procedure when a cost component is known to represent a large part of a total cost.

## Additional file

Additional file 1: Supplemental digital content file. (DOCX 29 kb)
