# Supplementary material for: Mixed method versus full top-down microcosting for organ recovery cost assessment in a French hospital group
Source: Health Econ Rev. 2016 Nov 28;6:53. doi: 10.1186/s13561-016-0133-3 (PMC5126031; doi:10.1186/s13561-016-0133-3)
Supplement: Additional file 1: — Supplemental digital content file. (DOCX 29 kb) [file 13561_2016_133_MOESM1_ESM.docx]

SUPPLEMENTAL DIGITAL CONTENT FILE

**RESULTS**

| SDC, Table 1, Breakdown of organ recovery cost components | | | | | |
| --- | --- | --- | --- | --- | --- |
| Items | Surgery, Anesthesia,  Intensive Care | Logistics | Imaging | Biology | Consumables |
|  |  |  |  |  |  |
| **Sub-items** |  |  |  |  |  |
|  |  |  |  |  |  |
|  | Medical care staff | Sterilization | 2 EEG | HLA | Surgical kit |
|  | Permanent medical care | Biomedical engineering | + | Serology | + |
|  | Nursing staff | Hygiene | 1 AU | Viral genome | Preservation |
|  | Non-medical staff | Vigilance | + | Serology checkup | fluid (IGL) |
|  | Maintenance |  | 1 TR | Hematology checkup |  |
|  | Depreciation and cost of block occupation |  |  | Serum electrolytes |  |
|  |  |  | **Or** | ABO Blood-group |  |
|  |  |  |  | Blood gas analysis |  |
|  |  |  | 1 CA | Renal, liver, pancreas |  |
|  |  |  | + | and cardiac checkup |  |
|  |  |  | 1 TAP Scan |  |  |
|  |  |  |  |  |  |

EEG: Electroencephalography CA: Cerebral Angioscanner

AU: Abdominal Ultrasonography TAP Scanner: Thoraco Abdomino Pelvien Scanner

TR: Thoracic Radiography

**SDC, Table 2,** Cost of surgical kit for kidney recovery in the HCL (2011 Euros)

| **Identification** | **Quantification** | **Valuation**  **Unit Cost (€) Cost by surgical kit (€)** |  |
| --- | --- | --- | --- |
| Sterile jars | 2 | 57 114 |  |
| Y-tube | 2 | 1,31 2,62 |  |
| PLDS self-suture clamp | 1 | 156 156 |  |
| TA 90 self-suture clamp | 1 | 149 149 |  |
| GIA 80 self-suture clamp | 1 | 149 149 |  |
| Refill GIA 80 self-suture clamp | 1 | 118 118 |  |
| Refill TA 90 self-suture clamp | 1 | 95 95 |  |
| Standard clip applier | 1 | 59 59 |  |
| Short clip applier | 1 | 59 59 |  |
| Bard canulas | 4 | 40 160 |  |
| Powder compact | 4 | 0,04 0,16 |  |
| Biconical fitting | 2 | 0,37 0,74 |  |
| IGL (2L ; fluid preservation) | 4 | 364 1455 |  |
|  |  |  |  |
| **TOTAL** |  | **2,516** |  |

**SDC, Table 3,** Cost of surgical kit for pancreas recovery in the HCL (2011 Euros)

| **Identification** | **Quantification** | **Valuation**  **Unit Cost (€) Cost by surgical kit (€)** |  |
| --- | --- | --- | --- |
| Sterile jars | 2 | 57 114 |  |
| Y-tube | 2 | 1,31 2,62 |  |
| PLDS self-suture clamp | 1 | 156 156 |  |
| TA 90 self-suture clamp | 1 | 149 149 |  |
| GIA 80 self-suture clamp | 1 | 149 149 |  |
| Refill GIA 80 self-suture clamp | 1 | 118 118 |  |
| Refill TA 90 self-suture clamp | 1 | 95 95 |  |
| Standard clip applier | 1 | 59 59 |  |
| Short clip applier | 1 | 59 59 |  |
| Bard canulas | 4 | 40 160 |  |
| Powder compact | 4 | 0,04 0,16 |  |
| Biconical fitting | 2 | 0,37 0,74 |  |
| IGL (2L ; fluid preservation) | 1 | 364 364 |  |
|  |  |  |  |
| **TOTAL** |  | **894** |  |

**SDC, Table 4**, Cost of surgical kit for liver recovery in the HCL (2011 Euros)

| **Identification** | **Quantification** | **Valuation**  **Unit Cost (€) Cost by surgical kit (€)** |  |
| --- | --- | --- | --- |
| Iced saline bag | 10 | 7 70 |  |
| Small bag | 5 | 5,7 28,5 |  |
| Y-tube | 4 | 2,3 9,2 |  |
| Venous cannula Ch 12 | 2 | 16 32 |  |
| Venous cannula Ch 14 | 2 | 16 32 |  |
| Venous cannula Ch 16 | 2 | 16 32 |  |
| Venous cannula Ch 22 | 2 | 16 32 |  |
| Venous cannula Ch 28 | 2 | 16 32 |  |
| Pediatric umbilical catheter | 2 | 4 8 |  |
| GIA Blue 80 | 1 | 138 138 |  |
| Gia Refills 80 blue | 3 | 98,5 295 |  |
| Drain transcystic Ch 4 | 1 | 9,5 9,5 |  |
| Drain transcystic Ch 5 | 2 | 9,5 19 |  |
| Drain transcystic Ch 6 | 2 | 9,5 19 |  |
| Drain transcystic Ch 7 | 1 | 9,5 9,5 |  |
| Clip M | 1 | 61 61 |  |
| Clip S | 1 | 49 49 |  |
| Powder box ECBU | 3 | 0,05 0,15 |  |
| Portagerm | 3 | 1,3 3,9 |  |
| EDTA Tube | 3 | 0,06 0,18 |  |
| IGL (2L ; preservation fluid) | 1 | 364 364 |  |
| **TOTAL** |  | **1,244** |  |

| SDC, Table 5, Organs recovered in the HCL from January 2010 to December 2011 | | | |
| --- | --- | --- | --- |
| Organs removed | Donors after Brain Death | T N° of organs |  |
| *1 organ removed* |  |  |  |
|  | Kidneys alone | 23 | 23 |
|  | Liver alone | 5 | 5 |
|  | Pancreas alone | 0 | 0 |
|  | Other | 1 | 1 |
|  |  |  |  |
| 2 organs removed |  |  |  |
|  | Kidneys +Liver | 27 | 54 |
|  | Kidneys + Pancreas | 1 | 2 |
|  | Kidneys +Other | 1 | 2 |
|  | Other combination | 0 | 0 |
|  |  |  |  |
| *3 or more organs removed* |  |  |  |
|  | Kidneys + Liver + Pancreas + Other(s) | 23 | 89 |
|  | Other combination | 10 | 31 |
|  |  |  |  |
| Total |  | 91 | 207 |

**FIGURES AND LEGENDS**

**SDC, Table 1,** Breakdown of organ recovery cost components

**SDC, Table 2,** Cost of surgical kit for kidney recovery in the HCL (2011 Euros)

**SDC, Table 3,** Cost of surgical kit for pancreas recovery in the HCL (2011 Euros)

**SDC, Table 4**, Cost of surgical kit for liver recovery in the HCL (2011 Euros)

SDC, Table 5, Organs recovered in the HCL from January 2010 to December 2011
